# The Egas Moniz histology digital platform – a dream that came true

**DOI:** 10.1080/07853890.2021.1897395

**Published:** 2021-09-28

**Authors:** M. A. Cavacas, A. Pereira, V. Braz de Oliveira, M. Remédios, A. R. Cavacas, R. Gancho, F. Pimenta, J. Botelho, P. Henriques, G. Borrecho, P. Oliveira, J. Camisão, M. E. Marques, J. J. Mendes

**Affiliations:** a Centro de Investigação Interdisciplinar Egas Moniz (CiiEM), Egas Moniz Cooperativa de Ensino Superior, Caparica, Portugal; b Clinical Research Unit (CRU), Centro de Investigação Interdisciplinar Egas Moniz (CiiEM), Egas Moniz Cooperativa de Ensino Superior, Caparica, Portugal; c Egas Moniz Histology Department; d Egas Moniz Anatomy Department; e Egas Moniz Dentistry Student’s; f Egas Moniz MMD

## Abstract

**Introduction:**

Histology knowledge is important to medical sciences as well as an important support to understand the pathology. However, teachers and students feel the need for innovative methods for teaching normal histology [1]. The project of the Histology Egas Moniz digital platform was born to involve our students of several curricular years and to dynamise the teaching of Histology. We believe that the interaction between teachers and students should be enhanced. The younger generations have a set of digital tools that teachers should seek to accompany and encourage. All the mechanisms that result in benefits for the academic formation of our students must be supported as well. The challenge was launched, and I invited students from the first year to the last year and we started to work. Same of the co-workers are now MDs.

**Materials and methods:**

The samples were collected from our morphology laboratory. All the samples were processed to be observed by light microscopy. Then, the most didactic samples were selected and photographed. The identification and description of the photos were performed. Parallelly, we wrote the text about the main tissues that was included in the digital platform. We also prepared an interactive quiz with the questions and the answers to facilitate the study when our students are preparing for their exams. The existence of a team that has been working at long term moved by a common teaching pedagogical goal identified under the general mission of Egas Moniz and carrying its “DNA” for the future generations.

**Results:**

For the time being, the base structure of the digital platform has a technical sheet, 5 chapters dedicated to the main types of tissues and they are all illustrated with original photographs prepared by the work team. Also, a QUIZ with 50 original questions has been prepared, with the answer to each question given interactively.

**Discussion and conclusions:**

This is a pilot project inside our organisation and our study’s methodology, but it´s true that this kind of digital platforms have been performed in other Universities and seem to be a success [2]. We believe that the final product doesn`t have a stop because the dynamic is important, and the authors want to increase value and periodic improvements based on the student’s appreciations in the future. We believe that pedagogically it is a very relevant initiative that must continue its development.
